# Alcohol consumption modulates prelimbic cortex response to cocaine following sequential cocaine and alcohol polysubstance use in the rat

**DOI:** 10.3389/fphar.2023.1132689

**Published:** 2023-03-16

**Authors:** Javier R. Mesa, Emily Carter, Yasmin Padovan-Hernandez, Lori A. Knackstedt

**Affiliations:** ^1^ Department of Psychology, University of Florida, Gainesville, FL, United States; ^2^ Center for Addiction Research and Education, University of Florida, Gainesville, FL, United States; ^3^ Department of Psychology, Pennsylvania State University, University Park, PA, United States; ^4^ Solomon H. Snyder Department of Neuroscience, Johns Hopkins University, Baltimore, MD, United States

**Keywords:** ethanol, polydrug, sensitization, neurocircuitry, relapse

## Abstract

Polysubstance use (PSU), involves the consumption of more than one drug within a period of time and is prevalent among cocaine users. Ceftriaxone, a beta-lactam antibiotic, reliably attenuates reinstatement of cocaine seeking in pre-clinical models by restoring glutamate homeostasis following cocaine self-administration but fails to do so when rats consume both cocaine and alcohol (cocaine + alcohol PSU). We previously found that cocaine + alcohol PSU rats reinstate cocaine seeking similarly to cocaine-only rats, but demonstrate differences in reinstatement-induced c-Fos expression throughout the reward system, including a lack of change upon ceftriaxone treatment. Here, we used this model to determine if previous findings were caused by tolerance or sensitization to the pharmacological effects of cocaine. Male rats underwent intravenous cocaine self-administration immediately followed by 6 h of home cage access to water or unsweetened alcohol for 12 days. Rats subsequently underwent 10 daily instrumental extinction sessions, during which time they were treated with either vehicle or ceftriaxone. Rats then received a non-contingent cocaine injection and were perfused for later immunohistochemical analysis of c-Fos expression in the reward neurocircuitry. c-Fos expression in the prelimbic cortex correlated with total alcohol intake in PSU rats. There were no effects of either ceftriaxone or PSU on c-Fos expression in the infralimbic cortex, nucleus accumbens core and shell, basolateral amygdala, or ventral tegmental area. These results support the idea that PSU and ceftriaxone alter the neurobiology underlying drug-seeking behavior in the absence of pharmacological tolerance or sensitization to cocaine.

## Introduction

Cocaine use disorder (CUD) is a chronic, progressive condition marked by a strong tendency for relapse even after prolonged periods of abstinence. In 2020, approximately 1.3 million Americans met DSM-5 criteria for CUD (SAMHSA, 2022) and there are currently no FDA-approved medications to prevent cocaine relapse. It is possible that polysubstance use (PSU), the use of multiple drugs in a given time, confounds efforts to find effective therapies against cocaine relapse. Of persons with CUD, a substantial portion consume alcohol concurrently, sequentially, or simultaneously with cocaine (Kedia et al., 2007; Liu et al., 2018; Liu et al., 2019). Alcohol dependence may reduce the efficacy of CUD-treating medications in humans. For instance, alcohol co-use prevents modafinil from reducing cocaine seeking (Anderson et al., 2009). Thus, cocaine-alcohol PSU may promote unique neuroadaptations not seen in cocaine monosubstance use that undermine efforts to treat CUD.

Preclinical models of monosubstance use have identified important changes in glutamate dynamics that underlie both cocaine and alcohol seeking separately. Chronic cocaine self-administration dysregulates glutamate homeostasis in the nucleus accumbens (NA) core, which underlies relapse to cocaine seeking (McFarland et al., 2003; Kalivas, 2004). Two to 3 weeks of cocaine self-administration reduces local levels of basal glutamate (Trantham-Davidson et al., 2012). Yet, during reinstatement of cocaine seeking, regardless of reinstatement prime (cue, drug, footshock, context), NA core glutamate transmission increases and impeding this increase attenuates seeking behavior (McFarland et al., 2003; McFarland et al., 2004; LaCrosse et al., 2016; Smith et al., 2017). More precisely, this glutamate efflux arises from medial prefrontal cortical afferents, primarily from the prelimbic cortex (PL) (McFarland et al., 2003). Concordantly, chemogenetic and optogenetic inhibition of the PL to NA core pathway during cocaine- and cue-primed reinstatement consistently attenuates cocaine seeking (Stefanik et al., 2013; Kerstetter et al., 2016; Stefanik et al., 2016; Wright et al., 2018; Siemsen et al., 2022). Beyond changes in neurotransmission, chronic cocaine self-administration reduces NA core expression of the glutamate transporter GLT-1 (Knackstedt et al., 2010) and xCT (the catalytic subunit of the cysteine glutamate antiporter system xc-) (Baker et al., 2003; Knackstedt et al., 2010; Trantham-Davidson et al., 2012), both of which are critical for maintaining glutamate homeostasis within NA core. Various rodent models indicate less robust effects of alcohol consumption on NA core glutamate homeostasis but suggest that alcohol itself also dysregulates local glutamate dynamics. Following weeks of chronic intermittent alcohol vapor exposure, mice exhibit higher levels of nucleus accumbens extracellular glutamate (Griffin et al., 2014). Furthermore, several weeks of intermittent access to alcohol increases basal glutamate levels in NA core (Griffin et al., 2014) without altering GLT-1 (Pati et al., 2016) or xCT expression in outbred Sprague Dawley rats (Stennett et al., 2017). Some of these findings are more pronounced in alcohol preferring C57BL/6J mice, which exhibit increased glutamate efflux in NA core following repeated alcohol injections (Kapasova and Szumlinski, 2008).

Because NA core glutamate dysregulation underlies both cocaine and alcohol seeking, one potential treatment for both cocaine and alcohol use disorders is the beta-lactam antibiotic ceftriaxone. In rodents, ceftriaxone upregulates NA core expression of GLT1 and xCT and restores local glutamate reuptake following cocaine self-administration (Knackstedt et al., 2010; Trantham-Davidson et al., 2012), while decreasing cocaine seeking behaviors in cocaine-, cue- and context-primed reinstatement tests (Sari et al., 2009; Knackstedt et al., 2010; Trantham-Davidson et al., 2012; LaCrosse et al., 2016). Furthermore, ceftriaxone attenuates cue-primed reinstatement of alcohol seeking (Weiland et al., 2015), and decreases alcohol intake in male Sprague-Dawley rats (Stennett et al., 2017). However, following alcohol consumption, ceftriaxone upregulates xCT (Stennett et al., 2017), but not GLT1 (Pati et al., 2016), expression in the NA core.

Despite the prevalence of cocaine + alcohol PSU, most pre-clinical models of cocaine and alcohol use are monosubstance in design and PSU models are lacking. Using a rodent model of sequential cocaine + alcohol self-administration, we demonstrated that sequential cocaine + alcohol PSU (alcohol immediately following cocaine) induces unique neuroadaptations compared to either monosubstance cocaine or alcohol use. Specifically, in male Sprague-Dawley rats, cocaine + alcohol PSU results in increased NA core GLT-1 expression while cocaine monosubstance use decreases GLT-1 expression and alcohol has no effect (Stennett et al., 2020). The glutamate efflux that characterizes the reinstatement of cocaine seeking is no longer present during cocaine-primed and cue + cocaine-primed reinstatement in the PSU condition, despite equivalent degrees of reinstated cocaine-seeking (Stennett et al., 2020; Stennett and Knackstedt, 2020). Moreover, cue + cocaine-primed reinstatement-induced c-Fos expression differs between cocaine monosubstance and cocaine + alcohol PSU conditions. Namely, in the basolateral amygdala (BLA), greater c-Fos expression was observed in the cocaine + alcohol PSU condition compared to the cocaine-only condition. Within the NA core and shell and the PL and infralimbic (IL) cortices, cocaine-only rats displayed greater c-Fos expression than did PSU rats. In these regions, ceftriaxone reduced c-Fos expression only in the monosubstance condition and not in PSU. Within the ventral tegmental area (VTA), ceftriaxone increased c-Fos expression in monosubstance rats only and PSU rats demonstrated similar c-Fos levels to vehicle-treated cocaine-only rats.

At this time, it remains unclear whether tolerance or sensitization to the pharmacological effects of cocaine following a history of sequential cocaine + alcohol PSU underly the differences seen following a cue + cocaine-primed reinstatement test. For example, decreased c-Fos expression in the PSU condition in the IL and PL could arise from decreased cocaine-induced glutamate and/or dopamine release in these regions. We hypothesize that this is not the case, and that the PSU- and ceftriaxone-induced differences in c-Fos expression observed in our previous work were due to differences in the circuitry engaged during relapse and not tolerance or sensitization to the pharmacological effects of cocaine. Here, we test this hypothesis by assessing pharmacological tolerance or sensitization to non-contingent cocaine *via* quantification of c-Fos expression in the PL, IL, NA core, NA shell, BLA, and VTA of ceftriaxone and vehicle-treated rats that self-administered cocaine-only or cocaine followed sequentially by alcohol access in the homecage, exactly as in our previous work. C-fos is an immediate early gene that is produced upon stimulation of dopamine and glutamate receptors (Morelli et al., 1992; Wang, 1998). Rats in the present study were not permitted the opportunity to reinstate cocaine-seeking, thus allowing the assessment of the isolated effects of the cocaine-priming injection on c-Fos expression in the selected brain regions.

## Materials and methods

### Animals

8-week-old male Sprague Dawley rats (*n* = 41; Charles River Laboratories LLC, Raleigh, NC) were housed in a temperature-controlled vivarium and maintained on a 12 h reverse-light cycle. Only male rats were used to permit comparisons with our previous work on this topic, which also used male rats. All procedures and testing were carried out during the dark phase of the cycle. Animals were restricted to 20 g of standard rat chow daily and water was always provided *ad libitum*. All procedures were approved by the University of Florida’s Institutional Animal Care and Use Committee and followed the Guidelines of the Care and Use of Laboratory Animals.

### Drugs

Cocaine HCl was donated by the NIDA controlled substances program (Research Triangle Institute, NC) and dissolved in 0.9% physiological saline as a 4 mg/mL solution. Alcohol (Fisher Scientific, 100%) was diluted to 20% v/v with tap water. Ceftriaxone (Sigma-Aldrich, St. Louis, MO) was dissolved in 0.9% physiological saline vehicle and administered intraperitoneally (IP) at 200 mg/kg in 1 mL/kg.

### Surgeries

Rats underwent jugular catheterization surgeries following the intermittent access to alcohol period (described below). They were anesthetized using ketamine (87.5 mg/kg, IP) and xylazine (5 mg/kg, IP) administered at a volume of 1 mL/kg. One end of the catheter (SILASTIC tubing, ID 0.51 mm, OD 0.94 mm, Dow Corning, Midland, MI) was implanted into the jugular vein. The other end exited through a midscapular incision and attached to a cannula (Plastics One, Roanoake, VA) embedded in a harness (Instech, Plymouth Meeting, PA), which allowed for intravenous (IV) drug delivery.

### Intermittent access to alcohol and cocaine self-administration

**FIGURE 1:**
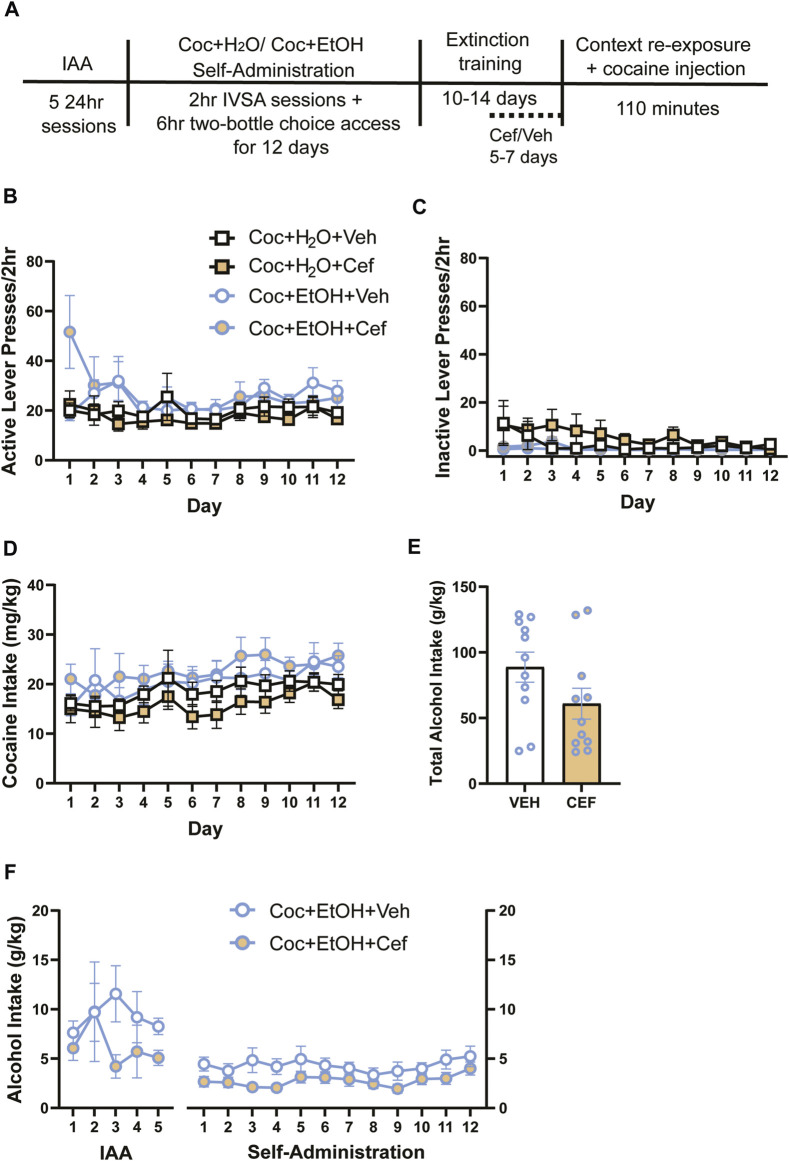
Sequential cocaine self-administration and subsequent two-bottle choice sessions. Access to alcohol does not alter intravenous cocaine self-administration variables, and rats later assigned to receive ceftriaxone and vehicle did not differ in cocaine or alcohol intake. **(A)** Timeline of methods. There were no differences in **(B)** active lever presses and **(C)** inactive lever presses across groups. **(D)** Cocaine intake did not vary between groups. Neither **(E)** alcohol intake during the IAA and cocaine self-administration periods nor **(F)** total alcohol intake differed in cocaine + alcohol PSU rats that received vehicle or ceftriaxone during extinction training.

To allow comparison between the present results and our previous study, we largely replicated the methods used in Stennett et al. (2020), with minor changes. Prior to the cocaine self-administration period, rats that were later in the cocaine + alcohol PSU condition were given intermittent access (IAA) to unsweetened alcohol for five 24-h sessions on alternating days (Figure 1A). All rats were then trained to self-administer intravenous cocaine for 2 h daily in a two-lever operant chamber. Active lever presses yielded a 0.1 mL cocaine infusion over 2.5 s and 5-s presentation of cues (2900 Hz tone and stimulus light) followed by a 20-s timeout period wherein additional active presses were recorded but did not yield programmed consequences. Inactive lever presses did not deliver cues or cocaine. Immediately following each cocaine self-administration session, the same rats that had previously received intermittent access to alcohol (*n* = 22) were presented with a two-bottle choice between water and unsweetened alcohol (20% v/v) in the home cage for 6 h, while the remaining rats (*n* = 19) received access to only water. Rats continued to self-administer cocaine and receive only water or the two-bottle choice until they administered at least 7 cocaine infusions per day for 12 days. One rat was excluded for failure to meet self-administration criteria and another for failed catheter patency.

### Cocaine extinction training and context re-exposure

Thereafter, rats underwent extinction training, wherein active lever presses did not yield cocaine infusions or corresponding cues. Rats did not receive access to either cocaine or alcohol during the extinction period. During the final 5–7 days of extinction, 21 rats (*n* = 11 PSU; *n* = 10 cocaine-only) were treated with ceftriaxone (200 mg/kg, IP) while 19 rats (*n* = 11 PSU; *n* = 8 cocaine-only) received vehicle.

To assess the pharmacological effects of non-contingent cocaine on neuronal activation, rats were re-exposed to their respective operant chambers for 110 min following an acute cocaine injection (10 mg/kg, IP). During this period, neither lever was extended and cocaine-associated cues were not presented. This time frame was selected to maximize c-Fos expression induced by the cocaine injection and follow our previous methods (Müller et al., 1984; Young et al., 1991; Mahler and Aston-Jones, 2012; Stennett et al., 2020).

### Immunohistochemistry

Immediately following the 110-min re-exposure, rats were euthanized and transcardially perfused using 4% PFA. Brains were extracted and preserved in 4% PFA for 24 h before transferring to 25% sucrose for 48 h. Brains were flash frozen using isopentane and stored at −80°C. Using a cryostat, tissue containing the PL, IL, NA core, NA shell, BLA, and VTA was coronally sliced at 30 µM and stored in phosphate-buffered saline with 0.01% sodium azide.

To visualize c-Fos expression, free-floating slices were blocked in 2% normal donkey serum and then incubated overnight in rabbit anti c-Fos antibody (1:10,000, EMD Millipore). Slices were then incubated in biotinylated donkey anti-rabbit secondary antibody followed by avidin-biotin complex (1:500, Vector Laboratories) and 3,3-diaminobenzidine (Vector Laboratories). Sections were mounted onto slides and coverslipped. Images were obtained at 20x using an AmScope MU1803 CMOS camera mounted to an Olympus BX51 microscope. Cellular c-Fos expression was quantified using NIH ImageJ software with a cell counter plug-in (DeVos, 2001).

### Statistical analysis

SPSS Statistics 28 was used to conduct 3-way ANOVAs and GraphPad Prism (9.4.1) was used to conduct 2-way ANOVAs, mixed effects, and correlational analyses. All analyses used an alpha level of 0.05. Mixed 3-way ANOVAs using Liquid (water or alcohol), and Treatment (vehicle or ceftriaxone) as between-subjects factors and Time as a within-subjects factor were used to compare active lever presses, inactive lever presses, infusions, and cocaine intake (mg/kg) across groups during the 12 days that self-administration criteria were met and to examine lever presses during extinction. Such analyses were used to balance ceftriaxone/vehicle treatment within cocaine and cocaine + alcohol groups. An unpaired *t*-test with Welch’s correction was also used to compare total alcohol intake (mg/kg) in cocaine + alcohol rats between treatment groups. c-Fos expression per mm^2^ was calculated and averaged between slices from the same region and rat and compared with 2-way Liquid x Treatment ANOVAs. Total cocaine intake was also compared with a 2-way ANOVA. Based on our *a priori* hypothesis that cocaine-alcohol PSU leads to increased tolerance to cocaine, we also conducted unpaired t-tests to compare c-Fos expression between cocaine-only and PSU rats that received vehicle. Outlier data was excluded when cell counts exceeded ≥2 standard deviations from sample means. c-Fos expression between groups was compared using 2-way ANOVAs with Liquid and Treatment as between-subjects factors. Spearman’s rank correlation was used to assess the strength of the relationship between total alcohol intake and total cocaine intake with c-Fos expression in regions of interest in cocaine + alcohol PSU rats.

## Results

### Cocaine self-administration, drug intake, and extinction

For cocaine self-administration variables, 3-way ANOVAs found no significant Liquid x Treatment x Time interactions, including for active lever presses (Figure 1B), inactive lever presses (Figure 1C), and cocaine intake (Figure 1D). In cases when Mauchly’s test of sphericity revealed a violation of the equal variances assumption, all epsilon (ε) values were below 0.75 and Greenhouse-Geisser corrections were used to reduce type I error. A main effect of Time on cocaine infusions was found [F_(5.872,211.386)_ = 8.459, *p* < 0.001], as infusions increased over time for all groups. Similarly, a main effect of Time on cocaine intake was found [F_(5.53,199.091)_ = 8.263, *p* < 0.001]. While there was no 3-way interaction for cocaine intake, there was a main effect of Liquid on cocaine intake [F_(1,36)_ = 6.271, *p* > 0.05]. To investigate this further, we summed the total cocaine intake (mg/kg) across all self-administration days and found no effects of Treatment or Liquid on cocaine intake (not shown). Of note, a mechanical failure on the first day of self-administration prevented recording of inactive lever presses for some rats (*n* = 10) that day. Rats that later received Vehicle and Ceftriaxone did not differ in the total amount of alcohol consumed throughout the experiment [t_{19.98)_ = 1.702, *p* > 0.05; Figure 1E]. We also found no Time x Treatment interaction and no main effect of Treatment on alcohol intake across the entire experiment (Figure 1F). When considering only the 12 days of cocaine-self-administration, we found that rats that later received Ceftriaxone during extinction had reduced alcohol intake [main effect of Treatment: F (1,15) = 4.547, *p* = 0.499].

**FIGURE 2:**
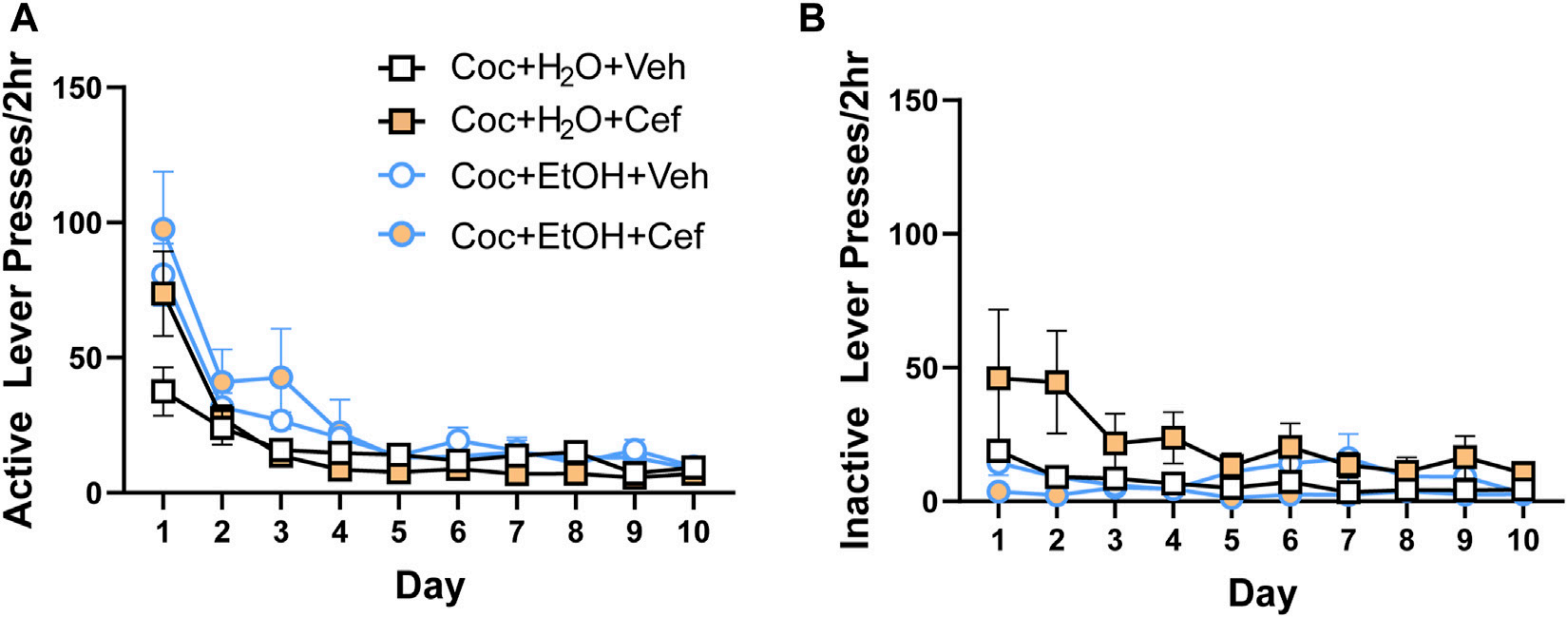
There were no group differences in lever presses during instrumental extinction. Both **(A)** active and **(B)** inactive lever presses decreased during extinction training, with no effects of liquid consumed or ceftriaxone/vehicle.

During extinction training, there were no Liquid x Treatment x Time interactions for presses on the previously active (Figure 2A) or inactive (Figure 2B) levers. Active and inactive lever presses decreased across all groups, evidenced by a main effect of Time on active lever presses [F_(1.98,71.285)_ = 43.624, *p* < 0.001] and inactive lever presses [F_(1.846,66.469)_ = 4.271, *p* > 0.05].

### c-Fos expression

**FIGURE 3:**
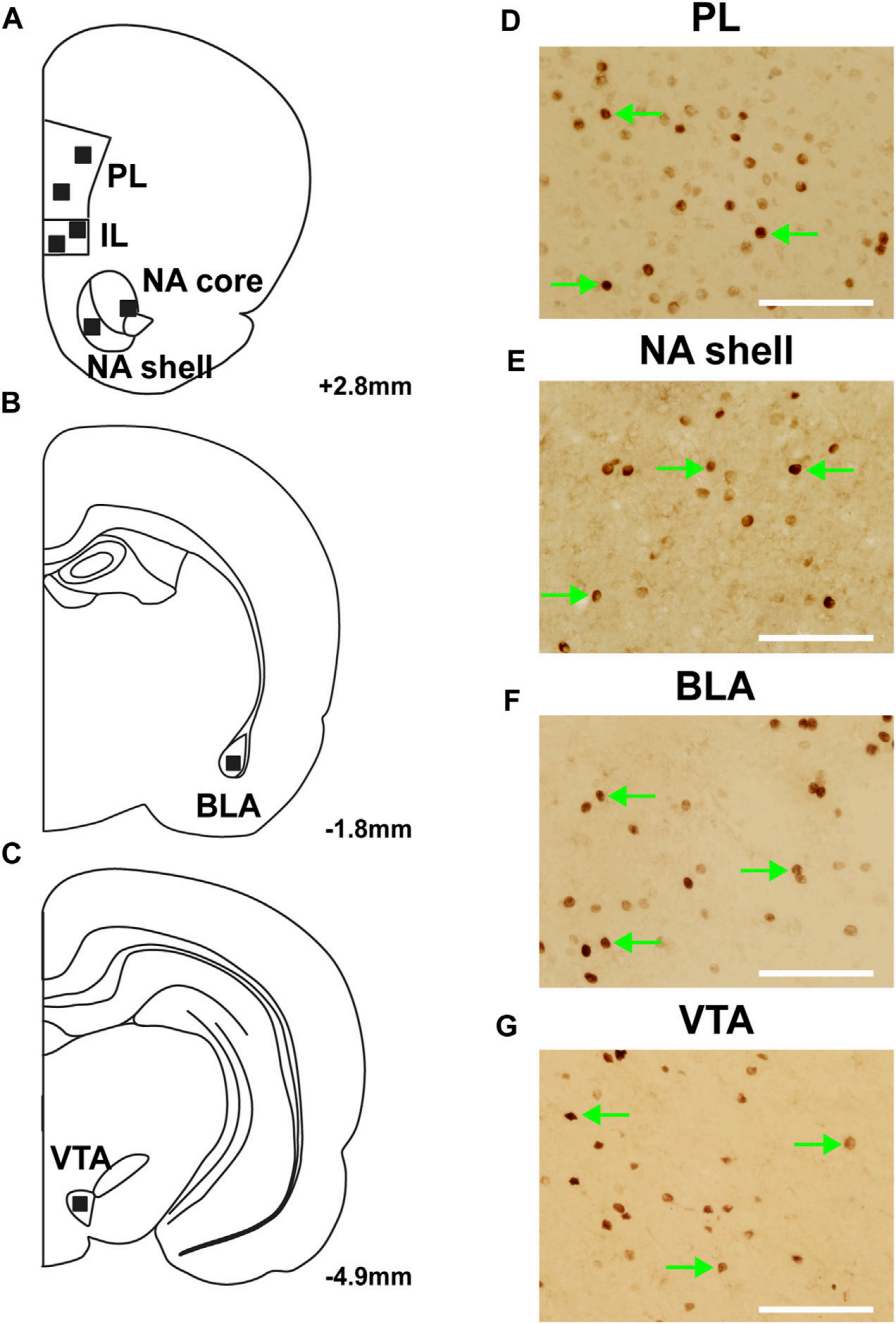
Representative images of c-Fos expression from regions of interest. **(A–C)** Location of analysis with corresponding coordinates relative to Bregma. Representative images of c-Fos expression in regions of interest: **(D)** prelimbic cortex (PL); **(E)** nucleus accumbens (NA) shell; **(F)** basolateral amygdala (BLA); and **(G)** ventral tegmental area (VTA). Green arrows represent c-fos + cells; scale bars = 100 µm.

Seven rats were excluded from c-Fos analysis due to poor perfusions. The regions of interest were determined using a rat brain atlas (Paxinos and Watson, 2007), and representative diagrams of those regions are depicted in Figures 3A–C. Sample images depicting c-Fos expression within the PL, NA shell, BLA and VTA are shown in Figures 3D–G.

**FIGURE 4:**
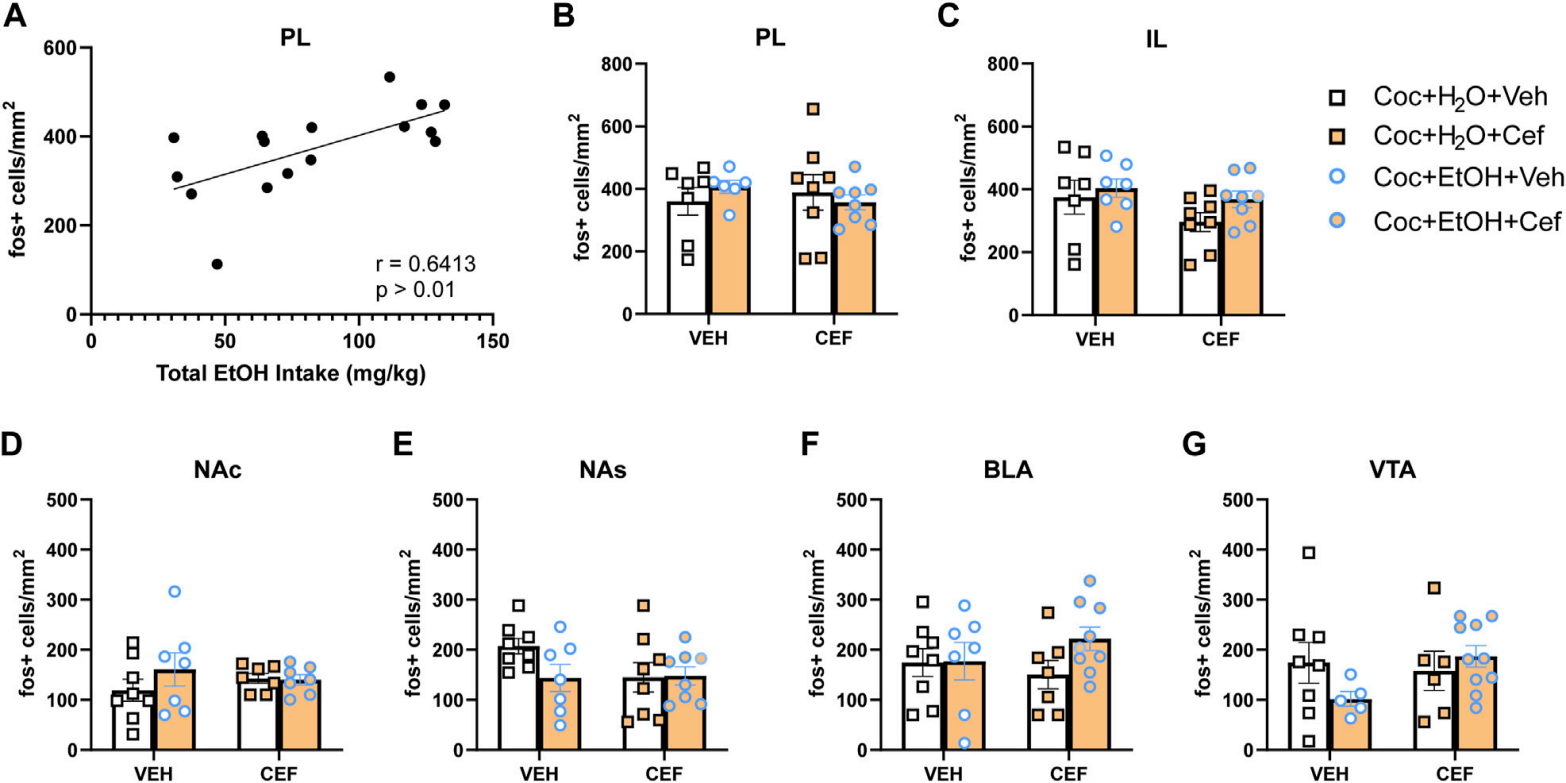
c-Fos expression induced by non-contingent cocaine injection and context re-exposure. **(A)** In cocaine + alcohol PSU rats, the total amount of alcohol intake positively correlated with c-Fos expression in the PL after a non-contingent cocaine injection (r = 0.6413, *p* > 0.01). c-Fos expression across groups in regions of interest: **(B)** PL; **(C)** IL; **(D)** NA core (NAc); **(E)** NA shell (NAs); **(F)** BLA; and **(G)** VTA.

There were no significant Treatment x Liquid interactions on c-Fos expression for any brain region examined. A positive correlation was found between total alcohol intake and PL c-Fos expression in cocaine + alcohol rats (r = 0.6413, *p* > 0.01; Figure 4A). No other significant correlations were detected between c-Fos expression and either cocaine or alcohol intake. To test the hypothesis that, independent of ceftriaxone treatment, PSU rats would display tolerance to the ability of cocaine to induce c-Fos, we conducted independent samples t-tests within cocaine-only and cocaine + alcohol vehicle-treated rats, finding no significant differences in c-Fos expression for any brain region (Figures 4B–G). However, there were trends for reduced c-Fos expression in the VTA [t_(10)_ = 1.944, *p* = 0.08; Figure 4E] and NAs [t_(13)_ = 2.093, *p* = 0.057; Figure 4G] of the cocaine + alcohol group.

## Discussion

Here we report the effects of a non-contingent cocaine injection on cellular activation in brain regions where we previously observed different levels of c-Fos expression upon a cue + cocaine-primed reinstatement test. We found no effects of either PSU or ceftriaxone treatment on cocaine-induced c-Fos expression in any brain region examined. In the PL, greater alcohol intake was associated with greater c-Fos expression upon non-contingent cocaine. We also report similar behavioral findings to our previous work using a sequential cocaine + alcohol model, with a modest increase in cocaine intake in the PSU condition. Previous iterations of the sequential cocaine + alcohol model did not produce detectable levels of cocaethylene, a psychoactive metabolite formed when alcohol and cocaine are both present in the liver, in samples of whole blood (Stennett et al., 2020). Thus, it is unlikely that cocaethylene affected our results.

We previously found that following a cue + cocaine-primed reinstatement test, cocaine + alcohol PSU rats demonstrate reduced c-Fos expression in the NA core, NA shell, and PL and IL cortices and increased expression in the BLA relative to cocaine monosubstance rats (Stennett et al., 2020). However, here, in the absence of cocaine-associated cues and the opportunity to relapse, a non-contingent cocaine injection produced c-Fos levels that were no different between cocaine + alcohol PSU and cocaine-only rats in these regions. While in the vehicle condition there were trends for reduced c-Fos expression in the VTA and the NA shell, these differences did not attain statistical significance. Thus, we can conclude that the differences in c-Fos expression observed previously after a cue + cocaine-primed reinstatement test resulted from a difference in neural circuitry mediating reinstatement in the PSU condition, and not tolerance or sensitization to cocaine. See Table 1 for summary of c-fos expression following non-contingent cocaine only and following a cue + cocaine-primed reinstatement test.

**TABLE 1:** C-fos expression in select brain regions following a cue + cocaine-primed reinstatement test (Stennett et al., 2020) or following only cocaine + context re-exposure (present data). Arrows refer to change in expression relative to the Coc + H_2_O + Veh condition.

Brain region	Stennett et al. (2020)				Present data		
	Coc+H_2_O-Cef	Coc+EtOH-Veh	Coc+EtOH-Cef		Coc+H_2_O-Cef	Coc+EtOH-Veh	Coc+EtOH-Cef
PL	↓	↓	↔		↔	↔	↔
IL	↓	↓	↔		↔	↔	↔
NA core	↓	↓	↓		↔	↔	↔
NA shell	↓	↓	↓		↔	↔	↔
BLA	↔	↑	↑		↔	↔	↔
VTA	↑	↔	↔		↔	↔	↔

The positive correlation between alcohol intake and the number of PL c-Fos + cells observed here suggests that alcohol consumption modulates PL responding to the pharmacological effects of cocaine. PL activity drives reinstatement of cocaine seeking (McFarland et al., 2003; McLaughlin and See, 2003; Stefanik et al., 2013; Mesa et al., 2022). If a non-contingent cocaine injection promotes cocaine seeking behaviors, PL cellular activation may serve as a precursor to those behaviors and alcohol may alter the capacity of those cells to respond to the cocaine experience. Pyramidal cells within PL undergo postsynaptic changes following chronic alcohol exposure, particularly in layers 2 and 3, from which efferents arise that terminate in the BLA (Gabbott et al., 2005). In mice, PL cells that project to the telencephalon (which contains the BLA) demonstrate greater spontaneous excitatory postsynaptic currents and mGlu_2/3_-dependent plasticity after 3 weeks of IAA (Joffe et al., 2021), a similar amount of alcohol exposure to that achieved here. Thus, acute cocaine use may act directly on excitatory PL neurons in layer 2/3 that were modified by chronic alcohol consumption, project to the BLA, and produce c-Fos. Of note, our study aimed to image layers 2/3 and 5 of the PL, the latter of which also sends projections to the BLA (Gabbott et al., 2005). Recent evidence, however, undermines the notion that alcohol exposure increases excitability in PL; intermittent alcohol vapor exposure did not alter intrinsic excitability of PL pyramidal neurons that project to either NA core or BLA and was accompanied by reduced expression of D1 receptors in that subpopulation (Obray et al., 2022). Yet, this work, like many other rodent studies on alcohol use, employed an intermittent alcohol exposure model, which tends to produce substantially higher levels of blood alcohol content than the sequential PSU model used here (Gass et al., 2014; Stennett et al., 2020). Thus, the effects of chronic alcohol on PL activation may be dose- and time-dependent. Cocaine intake leads to increased PL neuronal excitability (Nasif et al., 2005; Hearing et al., 2013; Otis et al., 2018). More work is needed to reconcile any competing effects of cocaine and alcohol on intrinsic excitability within the PL and the summation of such effects in PSU.

Our prior work found increased reinstatement-induced BLA c-Fos expression in the vehicle- and ceftriaxone-treated cocaine + alcohol PSU condition relative to the cocaine-only condition (Stennett et al., 2020). Here, we found no evidence that this arose from pharmacological sensitization to cocaine. Thus, the BLA may be a critical mediator of cue + cocaine-primed relapse in the PSU condition. It is well-established that regional inactivation of glutamatergic BLA cells impedes cue-primed reinstatement of cocaine seeking (Grimm and See, 2000; Kruzich and See, 2001; Kantak et al., 2002; Stefanik and Kalivas, 2013). The cytology of BLA suggests that c-Fos expression is likely present in glutamatergic principal neurons due to their relative abundance (McDonald, 1982; McCool, 2021), although c-Fos is also detectable in BLA GABAergic neurons as well (Berretta et al., 2005; Lukkes et al., 2012). Similarly, decreasing regional glutamate neurotransmission also demonstrably reduces alcohol seeking in rodent models (Bäckström et al., 2004; Gass et al., 2011; Sinclair et al., 2012). Future work will test the hypothesis that the BLA is necessary for reinstatement in the cocaine + alcohol condition.

Lastly, we previously found that cue + cocaine-primed reinstatement induces more VTA c-Fos in cocaine-only rats that are treated with ceftriaxone, an effect accompanied by decreased reinstatement. Here we do not see the same effect on c-Fos expression after non-contingent cocaine, indicating that our previous finding was unique to the cue + cocaine-primed reinstatement condition. Ceftriaxone does not have the same effect on reinstatement-induced c-Fos expression in the VTA of rats that consumed both cocaine and alcohol, nor does it attenuate reinstatement in this condition (Stennett et al., 2020).

## Conclusion

Here we find no evidence that sequential cocaine + alcohol PSU produces pharmacological tolerance or sensitization to cocaine in any brain region examined. Sequential cocaine + alcohol PSU increases PL responding to non-contingent cocaine, even in the absence of cocaine-associated cues. Future studies should aim to identify the profiles of PL cells altered by PSU as opposed to monosubstance use. The PL shares reciprocal projections with the BLA, a region that also experiences alcohol-related adaptations. Manipulation of BLA-PL pathways may help further define the changes caused by cocaine + alcohol PSU and how those changes drive seeking of each substance individually. To that end, additional research is needed to determine the necessity of this circuitry in the reinstatement of cocaine-seeking in a cocaine + alcohol PSU model. This work should include females, as all prior work on cocaine + alcohol PSU in rodents has been conducted in males.

## Data Availability

The raw data supporting the conclusion of this article will be made available by the authors, without undue reservation.
